# A hierarchical kidney outcome using win statistics in patients with heart failure from the DAPA-HF and DELIVER trials

**DOI:** 10.1038/s41591-024-02941-8

**Published:** 2024-05-06

**Authors:** Toru Kondo, Pardeep S. Jhund, Samvel B. Gasparyan, Mingming Yang, Brian L. Claggett, Finnian R. McCausland, Paolo Tolomeo, Muthiah Vadagunathan, Hiddo J. L. Heerspink, Scott D. Solomon, John J. V. McMurray

**Affiliations:** 1 British Heart Foundation Cardiovascular Research Centre, University of Glasgow, Glasgow, UK; 2 Department of Cardiology, Nagoya University Graduate School of Medicine, Nagoya, Japan; 3 Late-Stage Development, Cardiovascular, Renal, and Metabolism, BioPharmaceuticals R&D, AstraZeneca, Gothenburg, Sweden; 4 Department of Cardiology, Zhongda Hospital, School of Medicine, Southeast University, Nanjing, China; 5 Cardiovascular Division, Brigham and Women’s Hospital, Harvard Medical School, Boston, MA USA; 6 Renal Division, Brigham and Women’s Hospital, Harvard Medical School, Boston, MA USA; 7 Department of Clinical Pharmacy and Pharmacology, University of Groningen, Groningen, the Netherlands

**Keywords:** Heart failure, Randomized controlled trials

## Abstract

Win statistics offer a new approach to the analysis of outcomes in clinical trials, allowing the combination of time-to-event and longitudinal measurements and taking into account the clinical importance of the components of composite outcomes, as well as their relative timing. We examined this approach in a post hoc analysis of two trials that compared dapagliflozin to placebo in patients with heart failure and reduced ejection fraction (DAPA-HF) and mildly reduced or preserved ejection fraction (DELIVER). The effect of dapagliflozin on a hierarchical composite kidney outcome was assessed, including the following: (1) all-cause mortality; (2) end-stage kidney disease; (3) a decline in estimated glomerular filtration rate (eGFR) of ≥57%; (4) a decline in eGFR of ≥50%; (5) a decline in eGFR of ≥40%; and (6) participant-level eGFR slope. For this outcome, the win ratio was 1.10 (95% confidence interval (CI) = 1.06–1.15) in the combined dataset, 1.08 (95% CI = 1.01–1.16) in the DAPA-HF trial and 1.12 (95% CI = 1.05–1.18) in the DELIVER trial; that is, dapagliflozin was superior to placebo in both trials. The benefits of treatment were consistent in participants with and without baseline kidney disease, and with and without type 2 diabetes. In heart failure trials, win statistics may provide the statistical power to evaluate the effect of treatments on kidney as well as cardiovascular outcomes.

## Abstract (web summary)

Post hoc analysis of the DAPA-HF and DELIVER trials reports on the approach of win statistics to evaluate the effect of dapagliflozin on a hierarchical composite kidney outcome in patients with heart failure.

## Main

In patients with heart failure, kidney function is a powerful independent predictor of future heart failure hospitalization and death, irrespective of left ventricular ejection fraction (LVEF)^1–4^. The natural history of heart failure is characterized by progressive worsening of the syndrome over time and this usually includes worsening of kidney function^3,5–7^. Kidney function also influences whether life-saving pharmacological treatments, including renin–angiotensin system blockers and mineralocorticoid receptor antagonists (MRAs), can be initiated and continued in patients with heart failure and it determines eligibility for transplantation and mechanical circulatory support^8–18^. It is therefore important to understand the effect that new therapies for heart failure have on kidney function; an aspiration with any treatment for heart failure is to at least preserve and, ideally, improve kidney function.

Unfortunately, few trials in patients with heart failure have been large enough and long enough to accrue a sufficient number of ‘hard’ kidney endpoints to allow a statistically robust evaluation of these outcomes using conventional statistical approaches, for example, time-to-first-occurrence of death, end-stage kidney disease (ESKD) or a large decline in estimated glomerular filtration rate (eGFR)^19–23^. The rate of decline over time (slope) in eGFR has been used as an alternative means of evaluating the effect of treatment on kidney function; however^24–26^, while statistically more powerful, this measure does not incorporate death or initiation of renal replacement therapy and the clinical relevance of small changes in eGFR slope have been questioned.

The use of hierarchical composite endpoints analyzed with win statistics may solve some of these problems by integrating death, relatively infrequent major kidney events (for example, ESKD), the occurrence of large changes in eGFR that are somewhat more frequent, and changes in the eGFR slope, with each of these components ordered in a hierarchy reflecting their clinical importance^27–29^. The hierarchical composite outcome created by this approach consists of components, all of which reflect the progression of kidney disease, and this endpoint is both clinically relevant and statistically powerful^30^.

In this post hoc study, we evaluated the effects of dapagliflozin on kidney function in patients with heart failure and reduced ejection fraction, and heart failure and mildly reduced or preserved ejection fraction^31,32^, using a hierarchical composite kidney outcome, analyzed using win statistics.

## Results

Of the 11,004 participants included in the Dapagliflozin and Prevention of Adverse Outcomes in Heart Failure (DAPA-HF) and Dapagliflozin Evaluation to Improve the Lives of Patients with Preserved Ejection Fraction Heart Failure (DELIVER) trials, 4,742 were enrolled in DAPA-HF and 6,262 in DELIVER. Participants were assigned equally to dapagliflozin (*n* = 5,503) or placebo (*n* = 5,501).

### Participants

**Extended Data Fig. 1:**
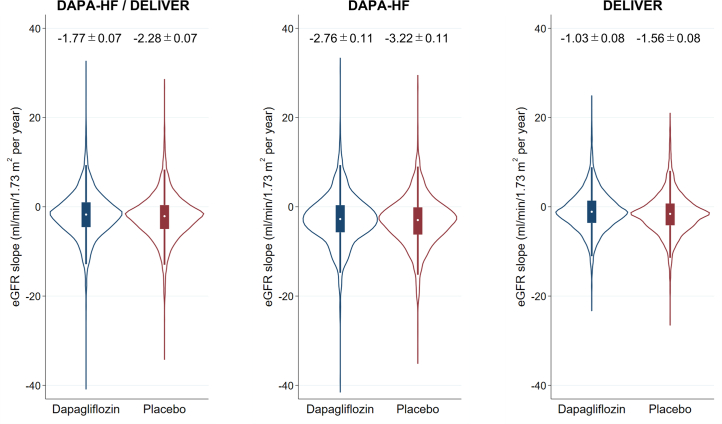
Distribution of the overall eGFR slope in each trial. The numbers above the violin plots are the mean±standard error of the eGFR slope. The white dots at the center of the violin plots show the median eGFR slope and the boxes around these indicate the 25th to 75th percentile values. The whiskers indicate the full range of the eGFR slope. The ‘violin’ shape is a density estimation showing the distribution of the eGFR slope values. Wider sections of the violin plot represent a higher probability and the narrower sections represent a lower probability of patients having the value. The eGFR slopes between randomized treatment groups are compared by the two-sided t-test. Adjustments are not made for multiple comparisons. The exact p-values were 0.0000002 in the pooled dataset, 0.004 in DAPA-HF, and 0.000004 in DELIVER. CI, confidence interval; DAPA-HF, Dapagliflozin and Prevention of Adverse Outcomes in Heart Failure; DELIVER, Dapagliflozin Evaluation to Improve the Lives of Patients with Preserved Ejection Fraction Heart Failure trial; eGFR, estimated glomerular filtration rate.

The participant characteristics according to the randomized treatment groups were well-balanced at baseline (Table 1). In the pooled dataset, there were 1,111 composite events of all-cause mortality, a decline of ≥40% in eGFR or ESKD or an eGFR <15 ml min^−1^ 1.73 m^−^^2^, in the dapagliflozin group, and 1,151 events in the placebo group; in the DAPA-HF trial, there were 458 in the dapagliflozin group and 509 in the placebo group; in the DELIVER trial, there were 653 in the dapagliflozin group and 642 in the placebo group (Table 2). The effects of dapagliflozin on conventional composite outcomes, analyzed as the time-to-first event, are shown in Table 2. In the pooled dataset, the total eGFR slope in the dapagliflozin group was significantly lower than in the placebo group (−1.77 ± 0.07 (mean ± s.e.) versus −2.28 ± 0.07 ml min^−1^ 1.73 m^−^^2^ per year, *P* < 0.001) (Table 2 and Extended Data Fig. 1). Similarly, in DAPA-HF and DELIVER separately, the total eGFR slope in the dapagliflozin group was significantly less steep than in the placebo group (DAPA-HF, −2.76 ± 0.11 (mean ± s.e.) versus −3.22 ± 0.11 ml min^−1^ 1.73 m^−^^2^ per year, *P* < 0.001; DELIVER, −1.03 ± 0.08 (mean ± SE) versus −1.56 ± 0.08 ml min^−1^ 1.73 m^−^^2^ per year, *P* = 0.004).

**Table 1:** Participant characteristics in the pooled DAPA-HF and DELIVER dataset

	Dapagliflozin	Placebo	*P*
	*n* = 5,503	*n* = 5,501	
Age, years	69.4 ± 10.6	69.4 ± 10.4	0.98
Female sex	1,928 (35.0)	1,927 (35.0)	>0.99
Region			0.96
Europe and Saudi Arabia	2,588 (47.0)	2,571 (46.7)	
North America	762 (13.8)	764 (13.9)	
South America	1,003 (18.2)	994 (18.1)	
Asia and the Pacific	1,150 (20.9)	1,172 (21.3)	
Ethnicity			0.40
White	3,875 (70.4)	3,894 (70.8)	
Asian	1,182 (21.5)	1,208 (22.0)	
Black or African American	203 (3.7)	182 (3.3)	
Other	243 (4.4)	217 (3.9)	
Baseline body mass index, kg m^−^^2^	29.1 ± 6.1	29.1 ± 6.1	0.99
**Vital signs**			
Heart rate, beats per min	71.5 ± 11.7	71.5 ± 11.7	0.98
Systolic blood pressure, mmHg	125.6 ± 16.1	125.4 ± 16.1	0.54
Diastolic blood pressure, mmHg	73.8 ± 10.4	73.7 ± 10.5	0.61
**Laboratory values**			
HbA1c, %	6.6 ± 1.4	6.5 ± 1.4	0.69
Creatinine, μmol l^−1^	103.0 ± 30.6	103.6 ± 31.0	0.28
eGFR, ml min^−1^ 1.73 m^−2^	63.2 ± 19.4	62.9 ± 19.4	0.35
eGFR < 60 ml min^−1^ 1.73 m^−2^ (%)	2,478 (45.0)	2,518 (45.8)	0.43
NT-proBNP, ng l^−1^	1,178 (708–2,129)	1,178 (698–2,117)	0.63
NT-proBNP, ng l^−1^ if baseline ECG in AF/AFL	1,569 (1,034–2,534)	1,525 (1,033–2,481)	0.49
NT-proBNP, ng l^−1^ if baseline ECG not in AF/ AFL	970 (563–1,820)	961 (566–1,846)	>0.99
AF/AFL on ECG (%)	1,896 (34.5)	1,875 (34.1)	0.68
**Heart failure characteristics**			
Previous hospitalization due to heart failure	2,393 (43.5)	2,395 (43.5)	0.96
Time from diagnosis of heart failure			0.29
≤1 year	1,520 (27.6)	1,579 (28.7)	
1–5 years	2,173 (39.5)	2,181 (39.7)	
>5 years	1,807 (32.9)	1,739 (31.6)	
NYHA functional class			0.097
I and II	3,919 (71.2)	3,995 (72.6)	
II and IV	1,584 (28.8)	1,505 (27.4)	
Baseline KCCQ-TSS	75.0 (56.3–89.6)	75.0 (58.3–89.6)	0.40
Baseline LVEF (%)	44.2 ± 13.7	44.2 ± 14.1	0.75
**Clinical history**			
T2D	2,394 (43.5)	2,395 (43.5)	0.97
AF	2,627 (47.7)	2,655 (48.3)	0.58
Hypertension	4,516 (82.1)	4,558 (82.9)	0.27
Myocardial infarction	1,830 (33.3)	1,900 (34.5)	0.15
Stroke	524 (9.5)	539 (9.8)	0.62
**Medical therapy**			
ACEi	2,476 (45.0)	2,480 (45.1)	0.93
ARB	1,807 (32.8)	1,769 (32.2)	0.45
ARNI	415 (7.5)	394 (7.2)	0.45
Beta-blocker	4,869 (88.5)	4,863 (88.4)	0.90
MRA	3,036 (55.2)	3,000 (54.5)	0.50
Loop diuretic	4,309 (78.3)	4,326 (78.6)	0.67
Digitalis	595 (10.8)	587 (10.7)	0.81
CRT-D or ICD	709 (12.9)	700 (12.7)	0.80

Data are presented as the mean ± s.d. or median (interquartile range) for continuous measures, and *n* (%) for categorical measures.

Continuous variables were compared using a two-sided *t*-test or Wilcoxon rank-sum test; categorical variables were compared using a chi-squared test. Adjustments for multiple comparisons were not made.

Body mass index was missing in eight participants; NT-proBNP was missing in one participant; AF/AFL on ECG was missing in two participants; time from diagnosis of heart failure was missing in five participants; and NYHA functional class was missing in one participant.

ACEi, angiotensin-converting enzyme inhibitor; AF, atrial fibrillation; AFL, atrial flutter; ARB, angiotensin receptor blocker; ARNI, angiotensin receptor neprilysin inhibitor; CRT-D, cardiac resynchronization therapy-defibrillator; ECG, electrocardiogram; HbA1c, glycated hemoglobin; ICD, implantable cardioverter-defibrillator; KCCQ-TSS, Kansas City Cardiomyopathy Questionnaire-Total Symptom Score; NT-proBNP, N-terminal pro-B-type natriuretic peptide; NYHA, New York Heart Association.

**Table 2:** Outcomes analyzed using conventional statistical approaches

	DAPA-HF and DELIVER					DAPA-HF					DELIVER				
	DAPA (*n* = 5,503)		Placebo (*n* = 5,501)		1/HR (95% CI)^a^	DAPA (*n* = 2,372)		Placebo (*n* = 2,370)		1/HR (95% CI)^a^	DAPA (*n* = 3,131)		Placebo (*n* = 3,131)		1/HR (95% CI)^a^
	Value	Rate per 100 patient-years (95 % CI)	Value	Rate per 100 patient-years (95% CI)		Value	Rate per 100 patient-years (95% CI)	Value	Rate per 100 patient-years (95% CI)		Value	Rate per 100 patient-years (95% CI)	Value	Rate per 100 patient-years (95% CI)	
Composite of all-cause mortality, ESKD or eGFR <15 ml min^−1^ 1.73 m^−^^2^, decline in eGFR ≥40%, *n*	1,111	15.7 (14.8–16.7)	1,151	16.2 (15.3–17.2)	1.04 (0.95–1.12)	458	15.0 (13.7–16.4)	509	16.8 (15.4–18.4)	1.12 (0.99–1.27)	653	16.3 (15.1–17.6)	642	15.7 (14.6–17.0)	0.98 (0.88–1.09)
Composite of all-cause mortality, ESKD or eGFR <15 ml min^−1^ 1.73 m^−^^2^, decline in eGFR ≥50%, *n*	884	12.2 (11.4–13.0)	979	13.5 (12.7–14.4)	1.11 (1.02–1.22)	330	10.5 (9.4–11.7)	402	13.0 (11.8–14.3)	1.24 (1.07–1.43)	554	13.5 (12.4–14.7)	577	13.9 (12.8–15.1)	1.04 (0.93–1.17)
All-cause mortality, *n*	769	7.5 (7.0–8.0)	847	8.3 (7.7–8.8)	1.11 (1.01–1.22)	275	8.0 (7.1–9.0)	326	9.6 (8.6–10.7)	1.20 (1.02–1.41)	494	7.2 (6.6–7.8)	521	7.6 (7.0–8.3)	1.06 (0.94–1.20)
ESKD or eGFR <15 ml min^−1^ 1.73 m^−^^2^, *n*	34	0.3 (0.2–0.5)	40	0.4 (0.3–0.5)	1.19 (0.75–1.88)	22	0.7 (0.4–1.0)	22	0.7 (0.4–1.0)	1.02 (0.56–1.83)	12	0.2 (0.1–0.3)	18	0.3 (0.2–0.4)	1.51 (0.73–3.13)
Decline in eGFR ≥57%, *n*	59	0.8 (0.6–1.0)	70	1.0 (0.8–1.2)	1.20 (0.85–1.69)	27	0.8 (0.6–1.2)	35	1.1 (0.8–1.5)	1.33 (0.80–2.19)	32	0.8 (0.5–1.1)	35	0.8 (0.6–1.2)	1.09 (0.67–1.75)
Decline in eGFR ≥50%, *n* (%)	132	1.8 (1.5–2.1)	147	2.0 (1.7–2.4)	1.12 (0.89–1.42)	61	1.9 (1.5–2.5)	83	2.6 (2.1–3.3)	1.39 (1.00–1.94)	71	1.7 (1.4–2.2)	64	1.5 (1.2–2.0)	0.89 (0.64–1.25)
Decline in eGFR ≥40%, *n*	398	5.6 (5.1–6.2)	356	5.0 (4.5–5.5)	0.90 (0.78–1.03)	202	6.5 (5.7–7.5)	208	6.8 (5.9–7.8)	1.04 (0.86–1.26)	196	4.9 (4.2–5.6)	148	3.6 (3.1–4.3)	0.75 (0.60–0.92)
eGFR slope, ml min^−1^ 1.73 m^−^^2^ per year^b^	−1.77 ± 0.07	–	−2.28 ± 0.07	–	–	−2.76 ± 0.11	–	−3.22 ± 0.11	–	–	−1.03 ± 0.08	–	−1.56 ± 0.08	–	–

^a^Models were stratified according to diabetes status (and according to trial in the pooled dataset).

^b^Data are presented as the mean ± s.e.

### Win ratio and proportion of wins and losses in each tier

**Fig. 1:**
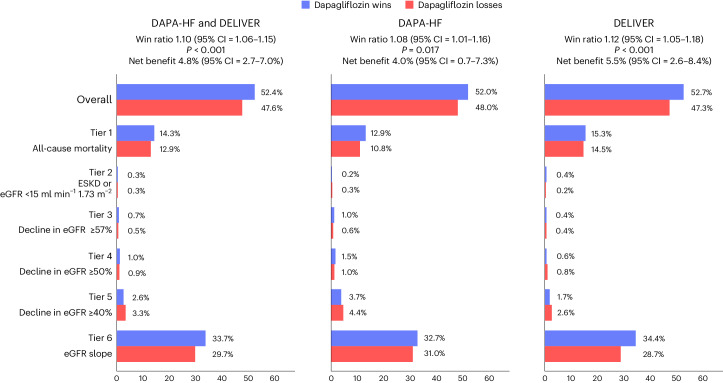
Effect of dapagliflozin on the hierarchical composite kidney outcome. Win statistics were two-sided. Models were stratified according to diabetes status (and according to trial in the pooled dataset). Adjustments were not made for multiple comparisons. The exact *P* values were 0.00001 in the pooled dataset and 0.0002 in the DELIVER dataset.

The effects of dapagliflozin on the hierarchical composite kidney outcome, as estimated using win statistics, are summarized in Fig. 1. The hierarchical composite kidney outcome included the following tiers: (1) all-cause mortality; (2) ESKD or eGFR <15 ml min^−1^ 1.73 m^−^^2^; (3) a decline in eGFR of ≥57%; (4) a decline in eGFR of ≥50%; (5) a decline in eGFR of ≥40%; and (6) participant-level eGFR slope. The win ratio was 1.10 (95% confidence interval (CI) = 1.06–1.15) in the pooled dataset, 1.08 (95% CI = 1.01–1.16) in DAPA-HF dataset and 1.12 (95% CI = 1.05–1.18) in the DELIVER dataset, demonstrating that dapagliflozin was superior to placebo with regard to the hierarchical composite kidney outcome compared in all three analyses. The eGFR slope accounted for most wins and losses, and incorporation of the participant-level eGFR slope in this model reduced the proportion of ties that would have occurred (in 63.4% of pairs in the pooled DAPA-HF and DELIVER dataset). The net benefit was 4.8% (95% CI = 2.7–7.0%) in the pooled dataset, 4.0% (95% CI = 0.7–7.3%) in the DAPA-HF dataset and 5.5% (95% CI = 2.6–8.4%) in the DELIVER dataset.

#### Sensitivity analyses

**Extended Data Fig. 2:**
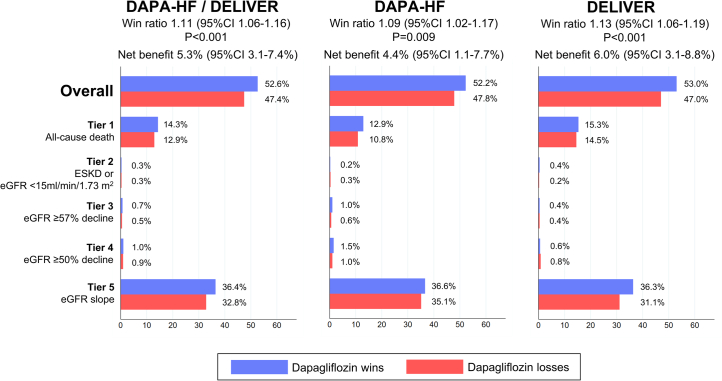
Effect of dapagliflozin on the hierarchical composite outcome in sensitivity model 1. Win statistics are two-sided. Models are stratified by diabetes status (and by trial in the pooled dataset). Adjustments are not made for multiple comparisons. The exact p-values were 0.000002 in the pooled dataset and 0.00005 in DELIVER. CI, confidence interval; DAPA-HF, Dapagliflozin and Prevention of Adverse Outcomes in Heart Failure; DELIVER, Dapagliflozin Evaluation to Improve the Lives of Patients with Preserved Ejection Fraction Heart Failure trial; eGFR, estimated glomerular filtration rate; ESKD, end-stage kidney disease.

**Extended Data Fig. 3:**
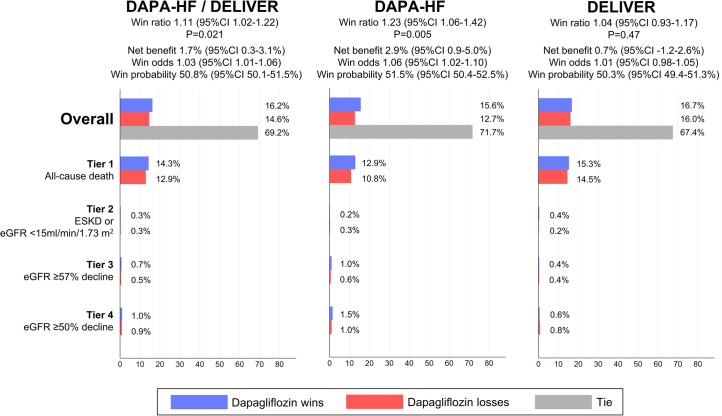
Effect of dapagliflozin on the hierarchical composite outcome in sensitivity model 2. Win statistics are two-sided. Models are stratified by diabetes status (and by trial in the pooled dataset). Adjustments are not made for multiple comparisons. CI, confidence interval; DAPA-HF, Dapagliflozin and Prevention of Adverse Outcomes in Heart Failure; DELIVER, Dapagliflozin Evaluation to Improve the Lives of Patients with Preserved Ejection Fraction Heart Failure trial; eGFR, estimated glomerular filtration rate; ESKD, end-stage kidney disease.

**Extended Data Fig. 4:**
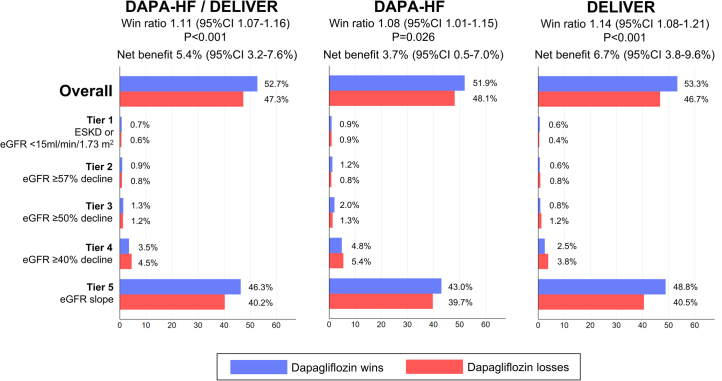
Effect of dapagliflozin on the hierarchical composite outcome in sensitivity model 3. Win statistics are two-sided. Models are stratified by diabetes status (and by trial in the pooled dataset). Adjustments are not made for multiple comparisons. The exact p-values were 0.0000009 in the pooled dataset and 0.000005 in DELIVER. CI, confidence interval; DAPA-HF, Dapagliflozin and Prevention of Adverse Outcomes in Heart Failure; DELIVER, Dapagliflozin Evaluation to Improve the Lives of Patients with Preserved Ejection Fraction Heart Failure trial; eGFR, estimated glomerular filtration rate; ESKD, end-stage kidney disease.

In the sensitivity Model 1 analysis, which excluded the tier for a decline in eGFR of ≥40%, win ratios remained higher than 1.0 for participants in the pooled dataset, and in the DAPA-HF and DELIVER trials separately (Extended Data Fig. 2). In sensitivity Model 2, which excluded both the tier for a decline in eGFR of ≥40% and the eGFR slope, the lower CIs of the win ratios and win odds (accounting for ties because of the exclusion of the eGFR slope) were not higher than 1.0 in the DELIVER dataset (Extended Data Fig. 3). The win ratios obtained using sensitivity Model 2 were similar to the 1/hazard ratios (HRs) for the composite kidney endpoints estimated using conventional statistical approaches and evaluated with the similar composite of all-cause mortality, ESKD or eGFR <15 ml min^−1^ 1.73 m^−^^2^, or decline in eGFR of ≥50% (Table 2). Adding the eGFR slope back into sensitivity Model 2 increased the net benefit from 1.7% to 5.3% in the pooled dataset. In sensitivity Model 3, which excluded all-cause mortality, almost identical results to the main model were observed in the pooled dataset, and the DAPA-HF and DELIVER datasets separately (Extended Data Fig. 4).

### Proportions of wins and losses over time

**Fig. 2:**
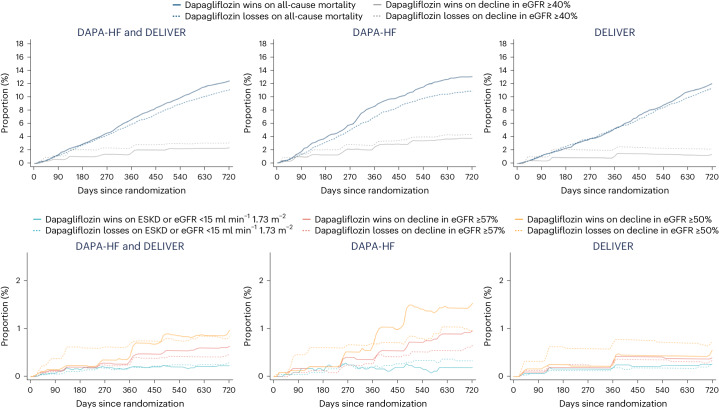
Proportion of wins and losses over time. Each figure was plotted every 10 days for up to 720 days.

**Extended Data Fig. 5:**
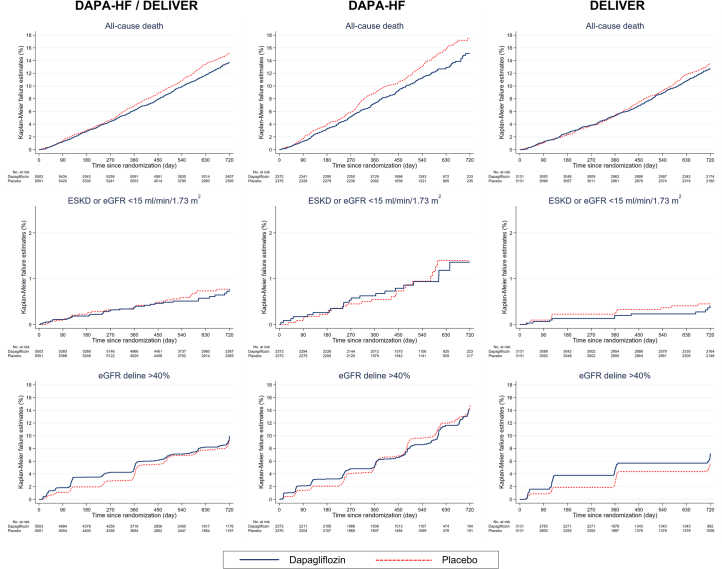
Effect of dapagliflozin in Kaplan-Meier plots. DAPA-HF, Dapagliflozin and Prevention of Adverse Outcomes in Heart Failure; DELIVER, Dapagliflozin Evaluation to Improve the Lives of Patients with Preserved Ejection Fraction Heart Failure trial; eGFR, estimated glomerular filtration rate; ESKD, end-stage kidney disease.

For all-cause mortality, differences in the proportion of wins and losses between treatments increased gradually over time in the pooled dataset, and in the DAPA-HF and DELIVER datasets separately (Fig. 2). In the three datasets, the proportion of losses with dapagliflozin for a decline in eGFR of ≥40% was larger than that of wins, but this difference narrowed over time. The proportions of wins and losses for ESKD or an eGFR <15 ml min^−1^ 1.73 m^−^^2^, and declines in eGFR of ≥57% and ≥50%, were small and differed little throughout the follow-up. For comparison, the effects of dapagliflozin versus placebo, plotted using the Kaplan–Meier method are shown in Extended Data Fig. 5.

### Win ratio and proportions of wins and losses in the subgroups

**Fig. 3:**
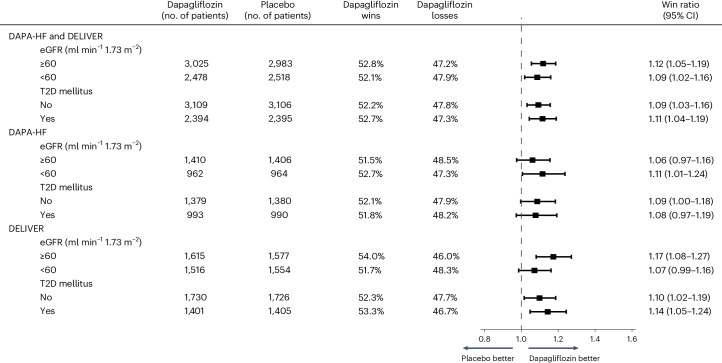
Effect of randomized treatments on the hierarchical composite kidney outcome according to selected subgroups. Models were stratified according to diabetes status (and according to the trial in the pooled dataset). The squares indicate the win ratios and the bars indicate the upper and lower boundaries of the 95% CI.

Win ratios, and the proportion of wins and losses, in the dapagliflozin groups according to a history of type 2 diabetes (T2D), eGFR category (<60 versus ≥60 ml min^−1^ 1.73 m^−^^2^) are shown in Fig. 3. The treatment effect estimate from the win ratio analysis was consistent across these subgroups, that is, there were no apparent differences in the estimates.

### Power analysis

**Extended Data Fig. 6:**
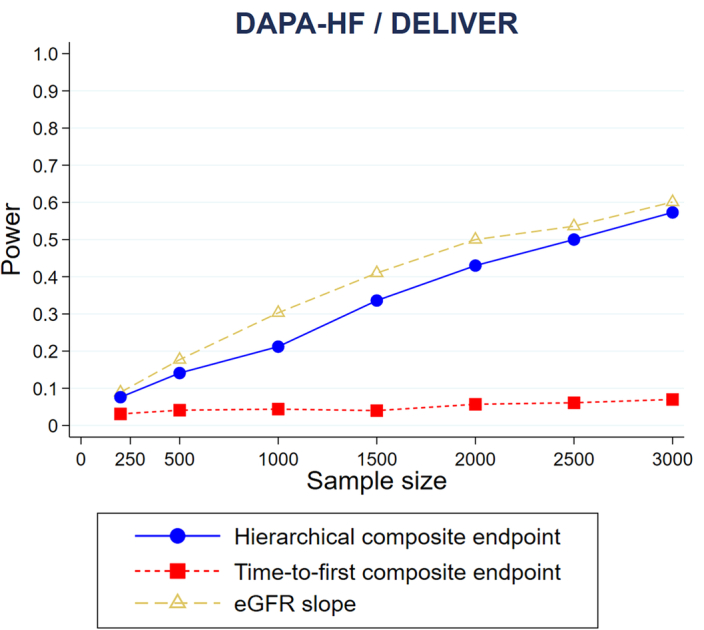
Curves for sample size and statistical power in the pooled dataset of DAPA-HF and DELIVER. DAPA-HF, Dapagliflozin and Prevention of Adverse Outcomes in Heart Failure; DELIVER, Dapagliflozin Evaluation to Improve the Lives of Patients with Preserved Ejection Fraction Heart Failure trial; eGFR, estimated glomerular filtration rate.

When using a hierarchical composite endpoint, sample size requirements are smaller than the time-to-first composite endpoint evaluated using the Cox proportional hazards model (Extended Data Fig. 6).

## Discussion

These post hoc analyses show how win statistics can be used to demonstrate the benefit of a treatment for heart failure (in this case, dapagliflozin) on kidney function in patients with both heart failure and reduced ejection fraction and heart failure and mildly reduced or preserved ejection fraction. It is generally difficult to demonstrate the potential kidney benefits of cardiovascular drugs using a conventional renal endpoint because of the small number of events in an ‘unenriched’ population (for example, without albuminuria) during a relatively short-term follow-up. In such a setting, the hierarchical composite endpoint examined in the present study provides greater statistical power and may offer the opportunity to demonstrate both cardiovascular and kidney benefits in the same population in the same trial. In addition to the summary of win statistics usually shown in analyses of this type, we also presented the proportion of wins and losses over time, similar to the depiction of event rates over time provided using traditional statistical methods.

Although superficially similar, the win statistics approach used in this study differs substantially from time-to-first-event analysis for a composite endpoint. The most obvious difference is that events are analyzed according to a hierarchy^27,28^. All-cause mortality was the most significant event in the composite hierarchical outcome and was tested as the first tier in the hierarchy. Unlike time-to-first-event analysis, the win statistics approach includes all deaths, including those occurring after a worsening kidney disease event. With the win statistics approach, a hierarchy of worsening kidney disease events was also created, reflecting their clinical importance, for example, the development of ESKD or an eGFR <15 ml min^−1^ 1.73 m^−^^2^, and large decreases in eGFR. As a further refinement, it is also possible to extend the hierarchy to include different proportional declines in eGFR; in the present analysis, we incorporated declines in eGFR of ≥57%, ≥50% and ≥40%. An additional advantage of win statistics is that the hierarchical composite outcome can logically incorporate continuous variables such as the eGFR slope^27–29^. Because the statistical power for conventional composite kidney outcomes is often insufficient when analyzing events such as those discussed above (because of their low incidence rate in some populations), analysis of the eGFR slope has been suggested as an alternative^19–22,24^. However, the eGFR slope is evaluated as a single ‘stand-alone’ outcome; its interpretation alongside other more important kidney endpoints simultaneously may not be easy. By contrast, the win statistics approach provides an outcome that integrates all relevant outcomes and all patients contribute to the analysis. One issue with the eGFR slope, either as a stand-alone endpoint or part of the win ratio approach, is that some drugs may cause an initial decline in eGFR^33–35^. The slope after initiation may more accurately reflect the chronic effect of these drugs, but may overestimate treatment benefit^36,37^; thus, more appropriately, we calculated the eGFR slope over the whole treatment period using a piece-wise, linear, two-slope model accounting for the effects of the acute and chronic phases^38^.

A closer look at the proportion of wins and losses revealed several findings. Despite the less steep eGFR slope with dapagliflozin compared to placebo, the proportion of wins with dapagliflozin (over placebo) for tier 5 of the hierarchy (that is, a decline in eGFR of ≥40%) was lower than the proportion of losses. The probable explanation for this is that DAPA-HF and DELIVER did not have an active run-in period and the initial drop in eGFR in some patients randomized to dapagliflozin led to a decline in eGFR counting as an ‘event’^31,32,39–42^. On examining the proportion of wins and losses over time, it can also be seen that the difference in the tier representing a decline in eGFR of ≥40%, which may reflect the initial drop with dapagliflozin early after randomization, was progressively smaller over time in the DAPA-HF and DELIVER, supporting this explanation and identifying the longer-term benefit of dapagliflozin on the kidney. Indeed the kidney benefits of both these drugs were more apparent over time, observed as the changing proportion of wins and losses over time, which is analogous to the divergence of Kaplan–Meier plots using conventional analysis.

Win statistics are a relatively new approach to analyzing trial data and may still be unfamiliar to some physicians^43,44^. However, their use is increasing rapidly, particularly in cardiovascular medicine; several recent trials had primary endpoints analyzed using win statistics^45–52^. At least one treatment has received regulatory approval based on a trial of this type^45^. Next, there is always debate about which components to include in a hierarchical composite outcome and these should be discussed between the relevant stakeholders, including patients, clinical trialists, and regulatory and reimbursement agencies. Although all-cause mortality is usually included as the first tier in such analyses, it could be argued that this is not a kidney-specific outcome^30^. To address this concern, we added a sensitivity analysis excluding all-cause mortality from the hierarchy, which showed essentially the same findings. Third, treatments may not affect each component of a composite outcome equally, although this is also an issue with composite endpoints evaluated using conventional statistics. Therefore, it is important to examine the proportion of wins or losses for each component of the composite to interpret the overall result.

This study has several limitations. eGFR was obtained at different scheduled visits in the two trials, while the incidence of the renal endpoints defined according to eGFR may have been affected by the frequency of the eGFR measurements. The hierarchical composite renal outcome used in this study was created post hoc. However, the selected hierarchy reflected the natural progression of kidney disease. It was validated in multiple sensitivity models and by comparison with the analysis of a conventional composite outcome analyzed using a standard method. The thresholds for declines in eGFR were also decided post hoc; thus, ‘sustained’ eGFR decline could not be confirmed using repeat measurement. The eGFR slope may also have been affected by the number of scheduled visits, visit intervals and the follow-up period in each trial.

In conclusion, it was possible to create a comprehensive, multicomponent, hierarchical composite kidney endpoint that is both clinically relevant and statistically powerful when analyzed using win statistics. With this approach, we confirmed the benefits of dapagliflozin on kidney function in patients with heart failure. This benefit was observed regardless of LVEF, baseline eGFR and T2D status. This approach can improve the power and precision around the estimate of effects on kidney outcomes and should be considered in future heart failure trials.

## Methods

### Study participants

In this post hoc study, we analyzed the DAPA-HF and DELIVER trials^31,32^. These were randomized, double-blind, placebo-controlled trials, and the trial designs and primary results have been published elsewhere^31,32,39–42^.

Briefly, DAPA-HF and DELIVER compared dapagliflozin to placebo in patients with a diagnosis of heart failure. Both trials enrolled patients with NYHA functional classes II–IV and elevated natriuretic peptide levels. The main difference between the two trials was that patients with an LVEF of ≤40% were randomized in the DAPA-HF trial and those with an LVEF >40% were randomized in the DELIVER trial. (DELIVER had evidence of structural heart disease, defined as either left atrial enlargement or left ventricular hypertrophy.) Key exclusion criteria included an eGFR lower than <30 ml min^−1^ 1.73 m^−^^2^ in DAPA-HF and an eGFR <25 ml min^−1^ 1.73 m^−2^ in DELIVER. In both trials, participants were randomized to receive dapagliflozin 10 mg once daily or a matching placebo. The median follow-up period was 1.5 years in the DAPA-HF trial and 2.3 years in the DELIVER trial.

Both trials were approved by the ethics committees at each investigative site and written informed consent was obtained from each participant.

### Study outcomes

The primary outcome was a composite of death from cardiovascular causes or worsening heart failure in DAPA-HF and DELIVER. In both trials, all-cause mortality was included as a secondary outcome, and a composite kidney outcome was included as a secondary outcome or prespecified exploratory outcome. All death events were adjudicated. The definition of ESKD in each trial was prespecified as a sustained eGFR <15 ml min^−1^ 1.73 m^−^^2^, chronic dialysis treatment or kidney transplantation in DAPA-HF and adverse event reporting, or a sustained eGFR <15 ml min^−1^ 1.73 m^−^^2^ in DELIVER. The endpoints driven by the eGFR were derived from central laboratory results.

In this post hoc analysis, we examined a hierarchical composite outcome including the following components: all-cause mortality (tier 1); ESKD or eGFR <15 ml min^−1^ 1.73 m^−^^2^ (tier 2); a decline in eGFR of ≥57% (tier 3); a decline in eGFR of ≥50% (tier 4); a decline in eGFR of ≥40% (tier 5); and participant-level eGFR slope (tier 6) (Extended Data Table 1). All-cause mortality was used for tier 1 in the hierarchy because of its ultimate clinical importance and its competing risk for the remaining outcomes. Considering the outcomes proposed by the international consensus definition of clinical trial outcomes for kidney disease, ESKD (or equivalent status) and decline in eGFR with different cutoffs were applied as tiers 2–5 (ref. ^53^). Decline in eGFR was applied as tier 6 because this has also been used for regulatory approval of treatment in some chronic kidney disease settings^24–26^. To address concerns regarding the lack of short-term verification of a change in eGFR due to the long interval between the scheduled study visits (and because some cutoffs were not verified as they were prespecified), declines in eGFR not requiring evidence that they were sustained eGFR were also evaluated. That is, change in eGFR (tiers 2–5) was evaluated as the time to the first meeting of the eGFR criterion based on the scheduled study visits, with the last laboratory assessment date used for censoring. eGFR was scheduled to be obtained at randomization, 14 days, 2 months, 4 months, 8 months, 12 months, 16 months, 20 months and 24 months in the DAPA-HF trial; and at randomization, 1 month, 4 months, 12 months, 24 months and 36 months in the DELIVER trial. The eGFR at randomization was used as the baseline eGFR to evaluate the change in eGFR; participants without baseline eGFR were excluded, that is, two participants in the DAPA-HF trial and one participant in the DELIVER trial. In this study, the original definition of ESKD in each study was used, alongside the aforementioned evaluation of change in eGFR.

As sensitivity analyses, we analyzed three additional models: sensitivity Model 1, excluding the component of a decline in eGFR of ≥40%, to evaluate outcomes less affected by the initial dip in eGFR due to the direct pharmacological action of dapagliflozin; sensitivity Model 2, excluding the component of a decline in eGFR of ≥40% and an eGFR slope to address additional concerns about the clinical relevance of the eGFR slope; and sensitivity Model 3, excluding all-cause mortality, which is more specific to kidney disease.

### Statistical analyses

To evaluate the effect of dapagliflozin across the range of LVEF, data were analyzed for the pooled dataset of DAPA-HF and DELIVER, and for each trial dataset separately.

Baseline characteristics were summarized according to the randomized group as the mean with s.d., or the median with the interquartile range for continuous variables and count with percentages for categorical variables. Continuous variables were compared using a *t*-test or Wilcoxon rank-sum test; categorical variables were compared using a chi-squared test. To determine the slope of change in eGFR for each individual patient over time according to the assigned treatment, two-slope, mixed-effect models accounting for the acute and chronic phases were applied using the eGFR data obtained at all scheduled visits^30^. The acute phase was defined as the period up to the first postrandomization visit (14 days in DAPA-HF and 1 month in DELIVER) when the acute treatment effect on the eGFR was considered fully present. These models were adjusted for baseline eGFR values, randomized treatment, visit time, diabetes status, spline variable corresponding to the days since the acute phase, the interaction of treatment and visit time, and the interaction of treatment and spline, without an intercept term. The distributions of the individual eGFR slopes were drawn using violin plots.

**Extended Data Fig. 7:**
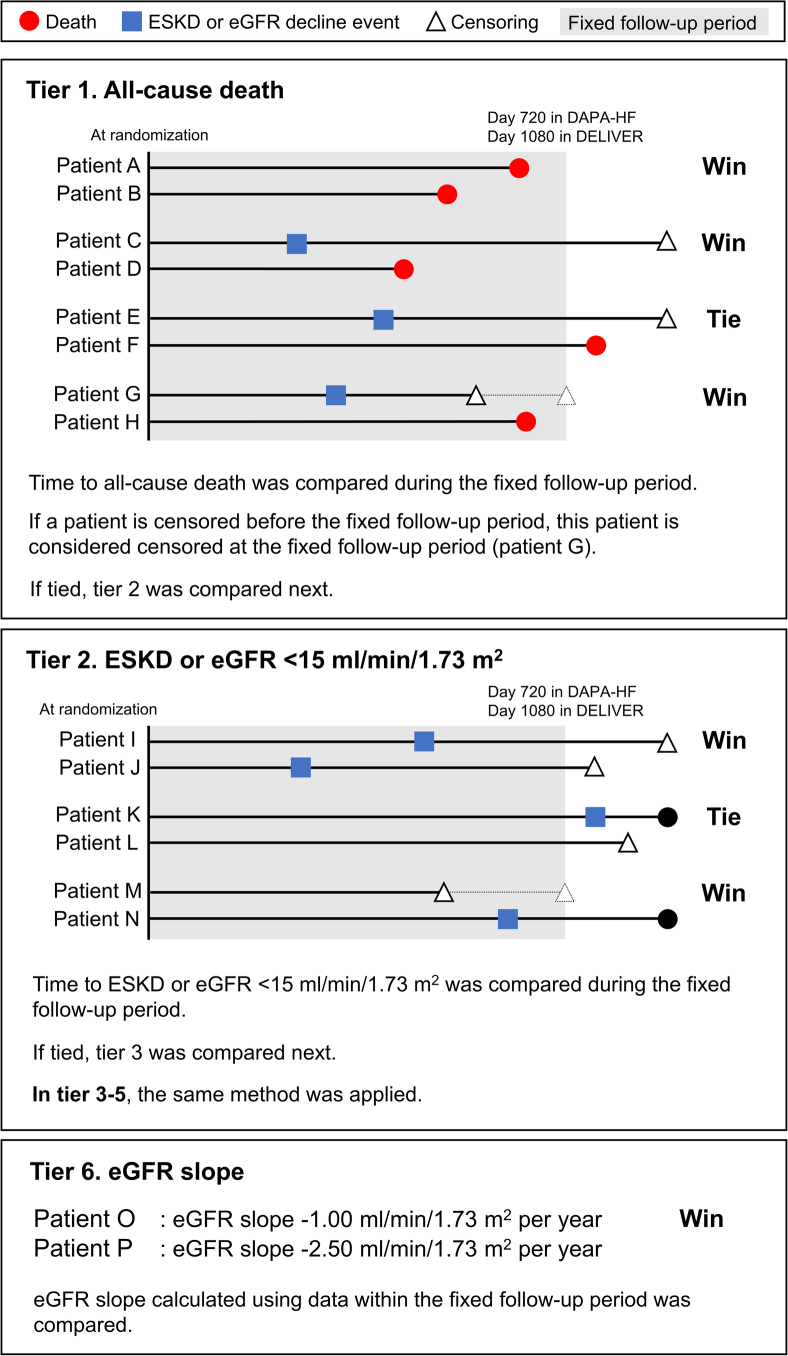
Testing the hierarchical composite outcome. DAPA-HF, Dapagliflozin and Prevention of Adverse Outcomes in Heart Failure; DELIVER, Dapagliflozin Evaluation to Improve the Lives of Patients with Preserved Ejection Fraction Heart Failure trial; eGFR, estimated glomerular filtration rate; ESKD, end-stage kidney disease.

The unmatched win statistics method, in which every patients in the dapagliflozin group was paired and compared with every patient in the placebo group, was used^27^; pairs representing the product of the number of individuals in the dapagliflozin group and placebo group were created and compared. Comparisons were made in ascending order of event tier (from 1 to 6); once a tier was settled, the next tier was not assessed; if the last tier was not settled, the comparison pair was considered a tie (Extended Data Fig. 7). In tiers 1–5, the time to first event was compared during a fixed follow-up period; censoring earlier than the defined fixed follow-up period was considered censoring at the fixed follow-up period to address the effect of censoring distributions on win statistics results^27,54–58^. Fixed follow-up periods were defined as 720 days in DAPA-HF and 1,080 days in DELIVER, considering the scheduled visits and follow-up period. In tier 6, the participant-level eGFR slope, which was calculated using data within these fixed follow-up periods, was compared as a continuous variable in each pair (that is, the patient with a shallower eGFR slope is the winner); thus, in the model including the eGFR slope, tied pairs did not exist. The proportions of win pairs (*P*_W_), loss pairs (*P*_L_) and tied pairs (*P*_T_) for participants assigned to dapagliflozin were obtained; *P*_W_ is the number of win pairs divided by the total number of pairs *n*_D_ × *n*_P_ where *n*_D_ and *n*_P_ are the sample sizes in the dapagliflozin and placebo group, similarly for *P*_L_ and *P*_T_. The method outlined by Pocock et al.^27^ and the corresponding variances based on the U-statistic-based method by Dong et al.^59^ were used to compute the win ratio. Because of a shortcoming of the win ratio that ignores ties when comparing pairs to obtain the win ratio, we calculated the ‘win odds’ for sensitivity Model 2, which is a modification of the win ratio accounting for ties^60,61^. Net benefit was also reported, which is the difference between the proportion of win and loss pairs^58^. We calculated four win statistics (win ratio, net benefit, win odds and win probability) defined as: win ratio, *P*_W_/*P*_L_; net benefit, *P*_W _− *P*_L_; win odds, (*P*_W_ + 0.5 *P*_T_)/(*P*_L_ + 0.5 *P*_T_); and win probability, *P*_W_ + 0.5 *P*_T_. Thus, in the main model, sensitivity Model 1 and sensitivity Model 3, where tied pairs do not exist, the win ratio is identical to the win odds. A win ratio represents the ratio of the proportion of win pairs to the proportion of loss pairs; a win rate greater than 1 with a lower 95% CI greater than 1 indicates that dapagliflozin is better than placebo. Because the win or loss proportion depends on the duration of follow-up and the censoring distribution, we plotted these trends over time every 10 days^55,62^. This plot was drawn only for tiers 1–5 because the eGFR slope was calculated based on data across the fixed follow-up period, meaning it was not possible to report an eGFR slope at a specific time point and illustrate the proportion of the wins or losses over time for this component of the composite outcome.

We also evaluated the component of the kidney hierarchical composite outcome up to the aforementioned fixed follow-up period using conventional statistical approaches to compare these results with the ones from the win statistic. Cox proportional hazards models were used to compute the HRs (to aid direct comparison, these are presented as 1/HR) and Kaplan–Meier curves were plotted.

Consistent with the prespecified stratification variables in each respective trial, win statistics and Cox proportional hazards models were stratified according to diabetes status and trial in the pooled dataset^31,32^.

The sample size requirements and statistical power of the hierarchical composite endpoint (main model) were compared using bootstrap resampling of the pooled dataset with the time-to-first composite endpoint (all-cause mortality, ESKD or eGFR <15 ml min^−1^ 1.73 m^−^^2^, or decline in eGFR of ≥40%) and eGFR slope to detect the observed treatment effect for each endpoint. The resampling procedure was performed with 1,000 iterations at each sample size (*n* = 200, 500 and increments of 500 until 3,000).

All analyses were conducted using STATA v.17.0 and R v.4.2.2.

### Reporting summary

Further information on research design is available in the Nature Portfolio Reporting Summary linked to this article.

## Online content

Any methods, additional references, Nature Portfolio reporting summaries, source data, extended data, supplementary information, acknowledgements, peer review information; details of author contributions and competing interests; and statements of data and code availability are available at 10.1038/s41591-024-02941-8.

### Supplementary information

Reporting Summary

## Figures and Tables

**Extended Data Table 1:**
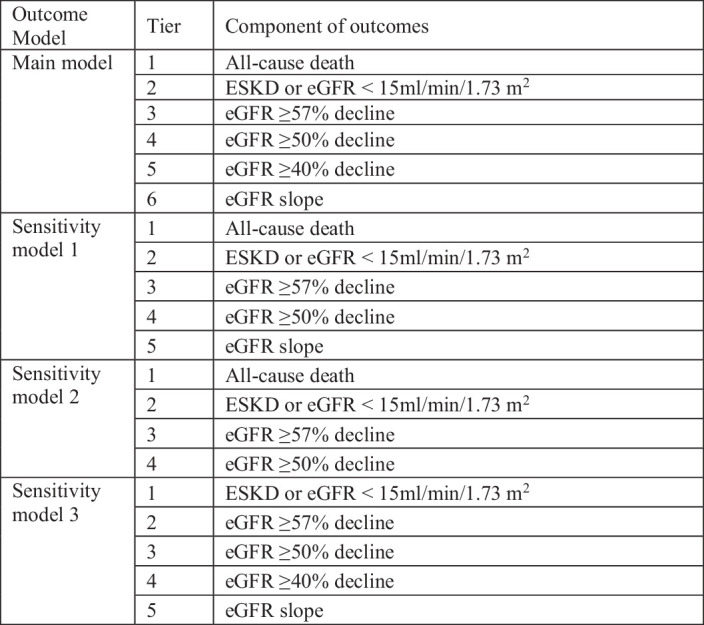
Summary of the hierarchical composite outcomes

eGFR, estimated glomerular filtration rate; ESKD, end-stage kidney disease.

## Data Availability

AstraZeneca’s data-sharing policy is described at https://astrazenecagrouptrials.pharmacm.com/ST/Submission/Disclosure. Researchers need to submit a request to access anonymized patient-level clinical data, aggregated clinical data or anonymized clinical study documents through Vivli’s web-based data request platform (https://vivli.org/). An independent scientific review board will review requests. Timelines vary per request and can take up to a year upon full submission of the request for analysis, decision, anonymization and sharing of the requested data or documents.

## Code availability

The key code to obtain the eGFR slope was published by Heerspink et al.^30^. Win statistics were conducted using the WINS package of R (https://cran.r-project.org/web/packages/WINS/index.html). The detailed code used to generate the findings of the present study is available from the corresponding author (john.mcmurray@glasgow.ac.uk) upon request from qualified researchers in this field. Researchers are asked to provide information on their affiliation and experience in this field and how they intend to use the code. The timelines vary per request and can take up to 6 months upon submission of the request.
