# Supplementary material for: A hierarchical kidney outcome using win statistics in patients with heart failure from the DAPA-HF and DELIVER trials
Source: Nat Med. 2024 May 6;30(5):1432–9. doi: 10.1038/s41591-024-02941-8 (PMC11108780; doi:10.1038/s41591-024-02941-8)
Supplement: Supplementary file 1 — Reporting Summary [file 41591_2024_2941_MOESM1_ESM.pdf]

Corresponding author(s): John JV McMurray

Last updated by author(s): Mar 5, 2024

Reporting Summary

Nature Portfolio wishes to improve the reproducibility of the work that we publish. This form provides structure for consistency and transparency in reporting. For further information on Nature Portfolio policies, see our Editorial Policies and the Editorial Policy Checklist.

Statistics

For all statistical analyses, confirm that the following items are present in the figure legend, table legend, main text, or Methods section.

n/a	Confirmed	
☐	☒	The exact sample size (n) for each experimental group/condition, given as a discrete number and unit of measurement
☐	☒	A statement on whether measurements were taken from distinct samples or whether the same sample was measured repeatedly
☐	☒	The statistical test(s) used AND whether they are one- or two-sided<br>*Only common tests should be described solely by name; describe more complex techniques in the Methods section.*
☐	☒	A description of all covariates tested
☐	☒	A description of any assumptions or corrections, such as tests of normality and adjustment for multiple comparisons
☐	☒	A full description of the statistical parameters including central tendency (e.g. means) or other basic estimates (e.g. regression coefficient) AND variation (e.g. standard deviation) or associated estimates of uncertainty (e.g. confidence intervals)
☐	☒	For null hypothesis testing, the test statistic (e.g. F, t, r) with confidence intervals, effect sizes, degrees of freedom and P value noted<br>*Give P values as exact values whenever suitable.*
☒	☐	For Bayesian analysis, information on the choice of priors and Markov chain Monte Carlo settings
☒	☐	For hierarchical and complex designs, identification of the appropriate level for tests and full reporting of outcomes
☒	☐	Estimates of effect sizes (e.g. Cohen's d, Pearson's r), indicating how they were calculated

Our web collection on statistics for biologists contains articles on many of the points above.

Software and code

Policy information about availability of computer code

Data collection	The key code to obtain the eGFR slope was published in the paper of Heerspink, H.J.L., et al. Development and Validation of a New Hierarchical Composite End Point for Clinical Trials of Kidney Disease Progression. J Am Soc Nephrol 34, 2025-2038 (2023). Win statistics were conducted using the WINS package of R (https://cran.r-project.org/web/packages/WINS/index.html).<br>The detailed code used to generate the findings of the present study is available from the corresponding author (john.mcmurray@glasgow.ac.uk) on request from qualified researchers in this field. Researchers are asked to provide information on their affiliation and experience in this field and how they intend to use the code. The timelines vary per request and can take up to 6 months upon submission of the request.
Data analysis	See above.

For manuscripts utilizing custom algorithms or software that are central to the research but not yet described in published literature, software must be made available to editors and reviewers. We strongly encourage code deposition in a community repository (e.g. GitHub). See the Nature Portfolio guidelines for submitting code & software for further information.

Data

Policy information about availability of data

All manuscripts must include a data availability statement. This statement should provide the following information, where applicable:

- Accession codes, unique identifiers, or web links for publicly available datasets
- A description of any restrictions on data availability
- For clinical datasets or third party data, please ensure that the statement adheres to our policy

AstraZeneca's data-sharing policy is described at https://astrazenecagrouptrials.pharmacm.com/ST/Submission/Disclosure.
Researchers need to submit a request to access anonymized patient-level clinical data, aggregated clinical data, and/or anonymized clinical study documents through Vivli's web-based data request platform (https://vivli.org/).
An independent Scientific Review Board is available to review requests.
The timelines vary per request and can take up to a year upon full submission of the request for analysis, decision, anonymisation and sharing of the requested data or documents.

Research involving human participants, their data, or biological material

Policy information about studies with human participants or human data. See also policy information about sex, gender (identity/presentation), and sexual orientation and race, ethnicity and racism.

Reporting on sex and gender	Biological sex was reported by investigators. Gender was not reported. Both sexes were enrolled (65% male and 35% female in this study). Thus, findings can be applied to both sexes. Analysis by sex was not performed because there is no difference in the effect of dapagliflozin by sex.<br><br>1: Wang X, Vaduganathan M, Claggett BL, Hegde SM, Pabon M, Kulac IJ, Vardeny O, O'Meara E, Zieroth S, Katova T, McGrath MM, Pouleur AC, Jhund PS, Desai AS, Inzucchi SE, Kosiborod MN, de Boer RA, Kober L, Sabatine MS, Martinez FA, Ponikowski P, Shah SJ, Hernandez AF, Langkilde AM, McMurray JJV, Solomon SD, Lam CSP. Sex Differences in Characteristics, Outcomes, and Treatment Response With Dapagliflozin Across the Range of Ejection Fraction in Patients With Heart Failure: Insights From DAPA-HF and DELIVER. Circulation. 2023 Feb 21;147(8):624-634. doi: 10.1161/CIRCULATIONAHA.122.062832. Epub 2022 Nov 7. PMID: 36342789; PMCID: PMC9974767.<br><br>2: Butt JH, Docherty KF, Petrie MC, Schou M, Kosiborod MN, O'Meara E, Katova T, Ljungman CEA, Diez M, Ogunniyi MO, Langkilde AM, Sjöstrand M, Lindholm D, Bengtsson O, Martinez FA, Ponikowski P, Sabatine MS, Solomon SD, Jhund PS, McMurray JJV, Køber L. Efficacy and Safety of Dapagliflozin in Men and Women With Heart Failure With Reduced Ejection Fraction: A Prespecified Analysis of the Dapagliflozin and Prevention of Adverse Outcomes in Heart Failure Trial. JAMA Cardiol. 2021 Jun 1;6(6):678-689. doi: 10.1001/jamacardio.2021.0379. PMID: 33787831; PMCID: PMC8014207.
Reporting on race, ethnicity, or other socially relevant groupings	Race was classified according to the United States Food and Drug Administration's guidance. Overall, 71% of patients were classified as White, 22% Asian, 3% Black or African-American, and 4% Other. Investigators were asked to report race as self-identified by patients. Race was not taken into account in the present analyses.
Population characteristics	HFrEF affects men more often than women (ratio of 2:1 or 3:1) whereas HFmrEF/HFpEF affects men and women similarly (or there is even a small female preponderance). The prevalence of both types of heart failure increases steeply with age but patients with HFmrEF/HFpEF are older, on average, than patients with HFrEF.
Recruitment	Recruitment was conducted at 410 sites in 20 countries (DAPA-HF) and at 353 sites in 20 countries (DELIVER) according to the protocol inclusion and exclusion criteria.
Ethics oversight	Both trials were registered at ClinicalTrials.gov before enrolment of the first patients, both protocols were approved by ethics committees at all sites, and all patients provided written informed consent, as described in the principal results manuscripts (see below):<br><br>1: McMurray JJV, Solomon SD, Inzucchi SE, Køber L, Kosiborod MN, Martinez FA, Ponikowski P, Sabatine MS, Anand IS, Bělohlávek J, Böhm M, Chiang CE, Chopra VK, de Boer RA, Desai AS, Diez M, Drozdz J, Dukát A, Ge J, Howlett JG, Katova T, Kitakaze M, Ljungman CEA, Merkely B, Nicolau JC, O'Meara E, Petrie MC, Vinh PN, Schou M, Tereshchenko S, Verma S, Held C, DeMets DL, Docherty KF, Jhund PS, Bengtsson O, Sjöstrand M, Langkilde AM; DAPA-HF Trial Committees and Investigators. Dapagliflozin in Patients with Heart Failure and Reduced Ejection Fraction. N Engl J Med. 2019 Nov 21;381(21):1995-2008. doi: 10.1056/NEJMoa1911303.<br><br>2: Solomon SD, McMurray JJV, Claggett B, de Boer RA, DeMets D, Hernandez AF, Inzucchi SE, Kosiborod MN, Lam CSP, Martinez F, Shah SJ, Desai AS, Jhund PS, Belohlavek J, Chiang CE, Borleffs CJW, Comin-Colet J, Dobreanu D, Drozdz J, Fang JC, Alcocer-Gamba MA, Al Habeeb W, Han Y, Cabrera Honorio JW, Janssens SP, Katova T, Kitakaze M, Merkely B, O'Meara E, Saraiva JFK, Tereshchenko SN, Thierer J, Vaduganathan M, Vardeny O, Verma S, Pham VN, Wilderäng U, Zaozerska N, Bachus E, Lindholm D, Petersson M, Langkilde AM; DELIVER Trial Committees and Investigators. Dapagliflozin in Heart Failure with

Mildly Reduced or Preserved Ejection Fraction. N Engl J Med. 2022 Sep 22;387(12):1089-1098. doi: 10.1056/NEJMoa2206286. Epub 2022 Aug 27. PMID: 36027570.

Note that full information on the approval of the study protocol must also be provided in the manuscript.

Field-specific reporting

Please select the one below that is the best fit for your research. If you are not sure, read the appropriate sections before making your selection.

☒ Life sciences ☐ Behavioural & social sciences ☐ Ecological, evolutionary & environmental sciences

For a reference copy of the document with all sections, see nature.com/documents/nr-reporting-summary-flat.pdf

Life sciences study design

All studies must disclose on these points even when the disclosure is negative.

Sample size

Sample size was 11007, 4744 from DAPA-HF and 6263 from DELIVER. The sample size and power calculations are described in the following:

1: McMurray JJV, Solomon SD, Inzucchi SE, Køber L, Kosiborod MN, Martinez FA, Ponikowski P, Sabatine MS, Anand IS, Bělohlávek J, Böhm M, Chiang CE, Chopra VK, de Boer RA, Desai AS, Diez M, Drozdz J, Dukát A, Ge J, Howlett JG, Katova T, Kitakaze M, Ljungman CEA, Merkely B, Nicolau JC, O'Meara E, Petrie MC, Vinh PN, Schou M, Tereshchenko S, Verma S, Held C, DeMets DL, Docherty KF, Jhund PS, Bengtsson O, Sjöstrand M, Langkilde AM; DAPA-HF Trial Committees and Investigators. Dapagliflozin in Patients with Heart Failure and Reduced Ejection Fraction. N Engl J Med. 2019 Nov 21;381(21):1995-2008. doi: 10.1056/NEJMoa1911303.

2: Solomon SD, McMurray JJV, Claggett B, de Boer RA, DeMets D, Hernandez AF, Inzucchi SE, Kosiborod MN, Lam CSP, Martinez F, Shah SJ, Desai AS, Jhund PS, Belohlavek J, Chiang CE, Borleffs CJW, Comin-Colet J, Dobreanu D, Drozdz J, Fang JC, Alcocer-Gamba MA, Al Habeeb W, Han Y, Cabrera Honorio JW, Janssens SP, Katova T, Kitakaze M, Merkely B, O'Meara E, Saraiva JFK, Tereshchenko SN, Thierer J, Vaduganathan M, Vardeny O, Verma S, Pham VN, Wilderäng U, Zaozerska N, Bachus E, Lindholm D, Petersson M, Langkilde AM; DELIVER Trial Committees and Investigators. Dapagliflozin in Heart Failure with Mildly Reduced or Preserved Ejection Fraction. N Engl J Med. 2022 Sep 22;387(12):1089-1098. doi: 10.1056/NEJMoa2206286. Epub 2022 Aug 27. PMID: 36027570.

Data exclusions

None.

Replication

Not reproducible because the data contains patient-specific information for over 11,000 cases

Randomization

Both were double blind placebo controlled prospective randomized trials.

Blinding

Both trials were double blind.

Reporting for specific materials, systems and methods

We require information from authors about some types of materials, experimental systems and methods used in many studies. Here, indicate whether each material, system or method listed is relevant to your study. If you are not sure if a list item applies to your research, read the appropriate section before selecting a response.

Materials & experimental systems

n/a	Involved in the study	
☒	☐	Antibodies
☒	☐	Eukaryotic cell lines
☒	☐	Palaeontology and archaeology
☒	☐	Animals and other organisms
☐	☒	Clinical data
☒	☐	Dual use research of concern
☒	☐	Plants

Methods

n/a	Involved in the study	
☒	☐	ChIP-seq
☒	☐	Flow cytometry
☒	☐	MRI-based neuroimaging

Clinical data

Policy information about clinical studies

All manuscripts should comply with the ICMJE guidelines for publication of clinical research and a completed CONSORT checklist must be included with all submissions.

Clinical trial registration

NCT03036124 and NCT03619213

Study protocol

Statistical plans have been submitted with manuscript. See also,

1: McMurray JJV, Solomon SD, Inzucchi SE, Køber L, Kosiborod MN, Martinez FA, Ponikowski P, Sabatine MS, Anand IS, Bělohlávek J,

Böhm M, Chiang CE, Chopra VK, de Boer RA, Desai AS, Diez M, Drozdz J, Dukát A, Ge J, Howlett JG, Katova T, Kitakaze M, Ljungman CEA, Merkely B, Nicolau JC, O'Meara E, Petrie MC, Vinh PN, Schou M, Tereshchenko S, Verma S, Held C, DeMets DL, Docherty KF, Jhund PS, Bengtsson O, Sjöstrand M, Langkilde AM; DAPA-HF Trial Committees and Investigators. Dapagliflozin in Patients with Heart Failure and Reduced Ejection Fraction. N Engl J Med. 2019 Nov 21;381(21):1995-2008. doi: 10.1056/NEJMoa1911303.

2: Solomon SD, McMurray JJV, Claggett B, de Boer RA, DeMets D, Hernandez AF, Inzucchi SE, Kosiborod MN, Lam CSP, Martinez F, Shah SJ, Desai AS, Jhund PS, Belohlavek J, Chiang CE, Borleffs CJW, Comin-Colet J, Dobreanu D, Drozdz J, Fang JC, Alcocer-Gamba MA, Al Habeeb W, Han Y, Cabrera Honorio JW, Janssens SP, Katova T, Kitakaze M, Merkely B, O'Meara E, Saraiva JFK, Tereshchenko SN, Thierer J, Vaduganathan M, Vardeny O, Verma S, Pham VN, Wilderäng U, Zaozerska N, Bachus E, Lindholm D, Petersson M, Langkilde AM; DELIVER Trial Committees and Investigators. Dapagliflozin in Heart Failure with Mildly Reduced or Preserved Ejection Fraction. N Engl J Med. 2022 Sep 22;387(12):1089-1098. doi: 10.1056/NEJMoa2206286. Epub 2022 Aug 27. PMID: 36027570.

Data collection

Patients were randomized between February 15, 2017, and August 17, 2018, at 410 centers in 20 countries in the DAPA-HF trial, and between August 27, 2018, and December 30, 2020, at 353 centers in 20 countries in the DELIVER trial. Data were analyzed independently at the University of Glasgow.

Outcomes

Both trials were event-driven and had the same primary endpoint which was a composite of the time to the first occurrence of worsening heart failure or death from a cardiovascular cause. Worsening heart failure was defined as unplanned hospital admission for heart failure or an urgent visit for worsening heart failure resulting in the administration of an intravenous diuretic.

In this post-hoc paper, we analyzed a hierarchical composite outcome including the following components: all-cause death (Tier 1); ESKD or eGFR <15ml/min/1.73 m2 (Tier 2); eGFR ≥57% decline (Tier 3); eGFR ≥50% decline (Tier 4); eGFR ≥40% decline (Tier 5); eGFR slope (Tier 6).

Plants

Seed stocks

N/A

Novel plant genotypes

N/A

Authentication

N/A
